# SGLT-2 inhibitors associated euglycemic and hyperglycemic DKA in a multicentric cohort

**DOI:** 10.1038/s41598-021-89752-w

**Published:** 2021-05-13

**Authors:** Fateen Ata, Zohaib Yousaf, Adeel Ahmad Khan, Almurtada Razok, Jaweria Akram, Elrazi Awadelkarim Hamid Ali, Ahmed Abdalhadi, Diaeldin Abdelgalil Ibrahim, Dabia Hamad S. H. Al Mohanadi, Mohammed I. Danjuma

**Affiliations:** 1 Department of Internal Medicine, Hamad General Hospital, Hamad Medical Corporation, PO BOX 3050, Doha, Qatar; 2 Division of Health Care Sciences, Dresden International University, Dresden, Germany; 3 Department of Geriatrics, Rumailah Hospital, Hamad Medical Corporation, Doha, Qatar; 4 Weill Cornell Medicine Qatar, Doha, Qatar; 5 Department of Endocrinology, Hamad General Hospital, Hamad Medical Corporation, Doha, Qatar; 6 College of Medicine, Qatar University, Doha, Qatar

**Keywords:** Diseases, Endocrinology

## Abstract

Euglycemic diabetic ketoacidosis (EuDKA) secondary to Sodium-glucose co-transporter-2 inhibitors (SGLT2i) in type 2 diabetes mellitus (T2D) is a rare but increasingly reported phenomenon. Not much is known about the burden of EuDKA in patients on SGLT2i or the associated factors. This retrospective cohort study tries to delineate the differences in factors associated with the development of EuDKA as compared to hyperglycemic DKA. We conducted a multicentre, retrospective study across three tertiary care centers under Weill Cornell affiliated-Hamad Medical Corporation, Qatar. The cohort comprised of T2D patients on SGLT2i who developed DKA between January 2015 to December 2020. The differences between the subjects who developed EuDKA or hyperglycaemic DKA (hDKA) were analyzed. A total of 9940 T2D patients were on SGLT2i during 2015–2020, out of which 43 developed DKA (0.43%). 25 developed EuKDA, whereas 18 had hDKA. The point prevalence of EuDKA in our cohort was 58.1%. EuDKA was most common in patients using canagliflozin, followed by empagliflozin and Dapagliflozin (100%, 77%, and 48.3%, respectively). Overall, infection (32.6%) was the most common trigger for DKA, followed by insulin non-compliance (13.7%). Infection was the only risk factor with a significant point estimate between the two groups, being more common in hDKA patients (p-value 0.006, RR 2.53, 95% CI 1.07–5.98). Canagliflozin had the strongest association with the development of EuDKA and was associated with the highest medical intensive care unit (MICU) admission rates (66.6%). In T2D patients on SGLT2i, infection is probably associated with an increased risk of developing EuDKA. The differential role of individual SGLT2i analogs is less clear and will need exploration by more extensive prospective studies.

## Introduction

SGLT2i is a relatively newer class of oral drugs for the management of T2D^1^. Their predominant mechanism of action lies in preventing glucose reabsorption from the proximal renal tubules by targeting the sodium-glucose transporter 2^2^. The most common adverse effect of SGLT2i is urinary tract infections^3^. A less common yet equally important side-effect associated with SGLT2i is DKA. SGLT-2 associated DKA is not an extensively studied phenomenon^4^.

DKA is more common in type 1 diabetes mellitus (T1D) than T2D (50–100 and 4.6–8 episodes per 1000 patients, respectively)^5,6^. However, it remains a significant cause of morbidity in the T2D population because of an overall higher prevalence of T2D itself (8.5% compared to 0.5% prevalence of T1D)^7^. DKA remains a significant healthcare burden with an estimated annual cost exceeding 1 billion USD^8^. DKA has been observed in T2D patients taking Glucagon-Like Peptide 1 Receptor (GLP-1) agonists and Dipeptidyl peptidase-4 (DPP-4) inhibitors^9,10^. However, the risk of DKA with SGLT2i is two to three times more than other oral T2D medications^4,10^.

EuDKA is a relatively new phenomenon that has most commonly been defined as ketoacidosis (pH ≤ 7.3, bicarbonate ≤ 15 mmol/L, anion gap > 12 mmol/L) in the presence of euglycemia (blood sugar < 14 mmol/L)^7^. The pathophysiology of DKA is the relative or absolute insulin-deficient state of the body^11^. Random blood sugar (RBS) testing is one of the easiest, rapid, readily available, and usually, the first test gives a real-time status of the blood sugar. This measurement is utilized to make a rapid diagnosis of DKA in home and hospital settings, irrespective of the healthcare system's resource status. However, RBS cannot be used as a “direct biomarker” for the body's insulin-deficient state in EuDKA. The RBS may misinform regarding the state of insulin deficiency in subjects using SGLT2i due to glucosuria leading to lower than anticipated serum glucose level despite a relative insulin-deficient state.

In 2015 FDA issued a warning regarding the risk of DKA with SGLT2i^12^. Clark et al. have recently provided the largest evidence of SGLT2i led DKA in the adult population^13^. Considering the emerging evidence, FDA in August 2020 released a recommendation to hold SGLT2i in diabetic patients before surgical procedures to avoid the development of DKA^14^. Due to the low prevalence of DKA (EuDKA and hDKA) in T2D patients taking SGLT2i, a comparative assessment of the two subsets is an area of interest. Often DKA of whatever phenotype (EuDKA or hDKA) are usually fraught with demonstrable morbidity and sometimes excess mortality. However, there has not been a comprehensive examination of the true burden and exact clinical phenotype of EuDKA in T2D patient cohorts on SGLT2i analogs. In this study, we conducted a retrospective examination on T2D patients on SGLT2i who developed DKA to ascertain the prevalence, demography, risk factors, and outcomes associated with the development of EuDKA.

## Methods

### Study design

We performed a cross-sectional, multicentre, retrospective study on T2D patients taking SGLT2i who developed either EuDKA or hDKA from January 2015 to December 2020.

### Inclusion criteria

All patients with T2D using SGLT2i and admitted to the three hospitals of Weill Cornell Medicine affiliated-Hamad Medical Corporation, Qatar, with diabetic ketoacidosis between 2015 and 2020 were included in the study. The patient cohort comprised patients who had a prior diagnosis of T2D based on HBA1C ≥ 47.5 mmol/mol (6.5%); fasting glucose ≥ 7.0; use of oral hypoglycaemic drugs with or without insulin use; patients were on one of the SGLT2i at the time of admission with DKA. The ketoacidosis diagnosis was made based on the following set of parameters: pH < 7.3, anion gap > 12 mmol/L, and ketonemia/ketonuria. Patients were further subdivided into EuDKA or hDKA based on the presence of either euglycemia (RBS < 14 mmol/L) or hyperglycemia (RBS ≥ 14 mmol/L). Patients who had DKA with T1D and those who had DKA with T2D but were not on SGLT2i were excluded from the study. Similarly, patients with T2D on SGLT2i admitted with a diagnosis of DKA who did not meet the definition of DKA (due to anion gap < 12) were excluded from the analysis (n = 3).

Data was abstracted from electronic records of Hamad Medical Corporation patient data repository (Cerner). Data collected includes demographics such as age, sex, ethnicity, body mass index (BMI), comorbid conditions, temperature, and relevant laboratory investigations at admission. Data related to patients’ diabetes status included HBA1c recorded on or 2–4 weeks before presentation, point of care (POC) glucose at admission, oral hypoglycaemic drugs, insulin use, concordance, and diabetic complications. The use and type of SGLT2i were confirmed from the medication histories and admission and discharge summaries of the included patients. Data collected for DKA includes relevant laboratory investigations such as PH, anion gap, lactic acid, POC glucose level, blood/urine ketones, possible triggering factors, DKA duration, hospital days, need for MICU admission, and in-hospital mortality.

### Statistical analyses

Descriptive and summary statistics were used to describe the study cohort’s socio-demographic parameters, with continuous variables presented as means (± SD) or median (interquartile range) as appropriate. In contrast, categorical variables were presented as numbers (percentages). The Shapiro–Wilk tests analyzed the normality of the data. Pairwise comparisons between continuous and categorical study variables were carried out with student t-test and chi-square tests, respectively. Kruskal–Wallis test and one way ANNOVA tests were used to compare the variables among the subgroups. We used a multiple linear regression model to analyze the associations of some of the more relevant independent variables with glucose at admission. These variables included fasting glucose within three months before admission (significant difference found in One-Way ANOVA), infections (significant difference found in Kruskal–Wallis test), diabetic retinopathy and compliance to insulin (reflecting the progression of diabetes and patient compliance to treatment), type of SGLT2i (to analyze any relation of different SGLT2i on glucose at admission). All data were analyzed using Jamovi version 1.2 (created in 2020, Sydney, Australia)^15^.

All methods were performed in accordance with the relevant guidelines and regulations and were in accordance with the Declaration of Helsinki. As this is a retrospective review of medical records, informed consent was waived by the Medical Research Center (MRC) Qatar.

### Ethics declaration

This work is original, has not been, and is not under consideration for publication in any other Journal. All authors have reviewed and approved the final version of the manuscript. The study was approved by the Medical Research Centre (MRC) Qatar (MRC-01-20-064).

### Consent to participate

Informed consent was not required, as this study was a retrospective data review of medical records.

### Consent for publication

Informed consent was not required, as this study was a retrospective data review of medical records.

## Results

### Baseline characteristics

Table 1 gives a summary of the patients’ baseline characteristics. During the five years (2015–2020), a total of 9940 T2D patients were on SGLT2i in the inpatient and outpatient settings of the three hospitals of Hamad Medical Corporation. Among these,43 developed DKA (prevalence: 0.43%). Out of 43 patients, 25 had EuDKA (prevalence: 0.25%) (10 males,15 females), and 18 had hDKA (prevalence: 0.18%) (9 males,9 females). The Mean age in EuDKA and hDKA was 52.4 (± 12.8) and 58.9 (± 12.9), respectively. There was a high proportion of patients from the Middle East-North Africa (MENA) region (n = 32; 20 EuDKA,12 hDKA). Mean BMI in EuDKA and hDKA were 30.2 (± 7.19) and 29.4 (± 6.17), respectively.

**Table 1:** Demographics, clinical characteristics, and outcomes of type 2 diabetes mellitus patients on SGLT2i who developed EuDKA or hDKA.

Baseline characteristics	Units	Total DKA (*N* = 43)	EuDKA (*N* = 25)	hDKA (*N* = 18)
Prevalence	NA	43/9940 (0.43%)	25/9940 (0.25%)	18/9940 (0.18%)
Age (Mean ± SD)	Years	55.1 ± 13.0	52.4 ± 12.8	58.9 ± 12.9
**Gender**	N (%)			
Male		19 (44.1%)	10 (40%)	9 (50%)
Female		24 (55.8%)	15 (60%)	9 (50%)
**Ethnicities**	N (%)			
MENA		32 (74.4%)	20	12
South-East Asian		6 (13.9%)	2	4
Others (Filipino, Australian)		5 (11.6%)	3	2
BMI (Mean ± SD)	kg/m^2^	29.8 ± 6.72	30.2 ± 7.19	29.4 ± 6.17
Fasting glucose (within last 3 months) (Mean ± SD)	mmol/L	10.6 ± 3.62	9.32 ± 2.86	12.6 ± 3.88
Random blood glucose at admission, Median (IQR)	mmol/L	12.2 (9.9–19.9)	10.3 (9.3–11.9)	21.8 (17.3–26.9)
Temperature, Median (IQR)	°C	36.8 (36.6–37)	36.7 (36.6–36.8)	36.9 (36.7–37.1)
HbA1c	mmol/mol	83.6	80.8	86.9
HbA1c (Mean ± SD)	Percentage	9.8 ± 1.95	9.54 ± 1.82	10.1 ± 2.12
White cell counts, Median (IQR)	10^3^/uL	11.1 (8.25–17.4)	11.1 (8.9–14.4)	11.7 (6.65–22.8)
Creatinine, Median (IQR)	umol/L	69 (52–106)	60 (52–85)	104 (17.1–130)
Lactate, Median (IQR)	mmol/L	1.45 (1.1–1.95)	1.4 (1–1.8)	1.6 (1.3–2.2)
Serum pH, Median (IQR)	NA	7.27 (7.14–7.32)	7.28 (7.16–7.32)	7.21 (7.07–7.32)
Anion Gap, Median (IQR)	mEq/L	20 (17.5–22.6)	19 (18–23)	20 (17.3–21.9)
Length of stay, Median (IQR)	Days	5 (3–9.5)	5 (4–12)	5 (3–7.75)
DKA duration, Median (IQR)	Days	2 (2–4)	2 (2–3)	2.5 (2–4)
**Triggering factors**	N (%)			
Infection		14 (32.6%)	4 (16%)	10 (55.5%)
Pancreatitis		2 (4.7%)	1 (4%)	1 (5.5%)
Surgery		1 (2.3%)	1 (4%)	0
Insulin non-compliance		4 (13.7%)	1 (6.25%)	3 (23%)
Unknown triggers		22 (51.1%)		
**Co-morbidities**	N (%)			
Coronary artery disease		8 (18.6%)	4 (16%)	4 (22.2%)
Heart failure		4 (9.3%)	3 (12%)	1 (5.5%)
Asthma		11 (25.6%)	6 (24%)	5 (27.7%)
Chronic liver Disease		1 (2.3%)	0	1 (5.5%)
Hypertension		23 (54.8%)	13 (52%)	10 (55.5%)
Malignancy		1 (2.3%)	1 (4%)	0
Psychiatric disorder		2 (4.6%)	0	2 (11.1%)
**Complications of diabetes**	N (%)			
Retinopathy		6 (14%)	4 (16%)	2 (11.1%)
Nephropathy		7(16.3%)	6 (24%)	1 (5.5%)
Diabetic Foot		2(4.7%)	1 (4%)	1 (5.5%)
Amputation		1(2.3%)	0	1 (5.5%)
Peripheral arterial disease		2(4.7%)	2 (8%)	0
**SGLT 2 inhibitors type**	N (%)			
Dapagliflozin		31 (72%)	15 (60%)	16 (88.8%)
Canagliflozin		3 (7%)	3 (12%)	0
Empagliflozin		9 (20.9%)	7 (28%)	2 (11.1%)
Current Insulin use	N (%)	29 (67.4%)	16 (64%)	13 (72%)
**Types of insulin**	N (%)			
Degludec		2 (4.7%)	2 (12.5%)	0
Glargine		11 (25.6%)	5 (31.25%)	6 (46.1%)
Aspart plus glargine		13 (30.2%)	9 (56.25%)	4 (30.7%)
Insulin pump		1 (2.3%)	0	1 (7.69%)
Lispro/Protamine		1 (2.3%)	0	1 (7.69%)
Mixtard		2 (4.7%)	1 (6.25%)	1 (7.69%)
Compliance to insulin	N (%)	25 (86.2%)	15 (93.75%)	10 (76.9%)
Sulfonylurea	N (%)	15 (34.9%)	10 (40%)	5 (27.7%)
Metformin	N (%)	28 (65.1%)	15 (60%)	13 (72.2%)
Thiazolidinediones	N (%)	15 (34.9%)	8 (32%)	7 (38.8%)
Meglitinides	N (%)	2 (4.7%)	0	2 (11.1%)
Alpha-glucosidase inhibitors	N (%)	1 (2.3%)	0	1 (5.5%0
GLP-1 agonist	N (%)	5 (11.6%)	3 (12%)	8 (44.4%)
DDP-4 inhibitors	N (%)	17 (39.5%)	9 (36%)	8 (44.4%)
Inhaled Corticosteroids	N (%)	11 (25.6%)	6 (24%)	5 (27.7%)
In-hospital mortality	N (%)	1 (2.3%)	1 (4%)	0
Need for admission to ICU	N (%)	19 (44.2%)	11 (44%)	8 (44.4%)

T2D: Type 2 diabetes mellitus, SGLT2i: Sodium-glucose co-transporter 2 inhibitors, MENA: Middle East and North Africa EuDKA: Euglycemic diabetic ketoacidosis, hDKA: Hyperglycaemic diabetic ketoacidosis, GLP-1: glucagon-like peptide-1, DPP-4: dipeptidyl-peptidase-4, ICU: intensive care unit, NA: Not applicable.

### Laboratory results and clinical outcomes in EuDKA and hDKA groups

Median RBS at admission was 10.3 (9.3–11.9) in EuDKA and 21.8 (17.3–26.9) in hDKA. The difference was statistically significant, as expected (p value < 0.001). Mean fasting glucose (measured within three months before admission) was 9.32 ± 2.86 in EuDKA and 12.6 ± 3.88 in the hDKA group (p value 0.023). Diabetic complications were more frequent in the EuDKA group compared to hDKA. The difference in the frequency of diabetic retinopathy ( 16% in EuDKA and 11.1% in hDKA) was statistically significant (p-value 0.02). Mean HbA1c was 80.8 mmol/mol (9.54% ± 1.82) in EuDKA and 86.9 mmol/mol (10.1% ± 2.12) in hDKA [Table 1]. EuDKA patients had a median pH of 7.28 (7.16–7.32), while hDKA had 7.21 (7.07–7.32). The median length of stay was five days in both groups (IQR 4–12 in EuDKA and 3–7.75 in hDKA). The median collective anion gap was 20 (17.5–22.6), with comparable results in both groups [Table 1]. Median DKA duration was 2 (IQR 2–3) days in EuDKA and 2.5 (IQR 2–4) days in hDKA. The intensive care unit admissions rate was similar in both groups [Table 1].

### Precipitating factors

Infection was the most common trigger overall (32.6%), followed by insulin non-compliance (13.7%), pancreatitis (4.7%), and surgery (2.3%); precipitating factors had a similar trend in both EuDKA and hDKA except for infections, which was more in hDKA patients compared to EuDKA (RR 2.53, 95% CI 1.07–5.98 p value 0.006). The difference remained significant after multiple linear regression analysis (p-value 0.039).

### Results based on SGLT2-i type

Among the total population on SGLT2i (9940 patients), the prevalence of total DKA, EuDKA, and hDKA in patients taking Dapagliflozin (7280 patients) was 0.43%, 0.21%, and 0.23%, respectively. The prevalence of total DKA, EuDKA, and hDKA in patients taking Empagliflozin (2646) was 0.34%, 0.26%, and 0.08%. Lastly, the prevalence of total DKA and EuDKA in patients taking Canagliflozin (14 patients) was 0.21%. The prevalence of hDKA in patients on Canagliflozin could not be calculated due to the small sample size. In our cohort of 43 T2D patients who developed DKA, the most common SGLT2i used was Dapagliflozin (72%), followed by empagliflozin (20.9%) and canagliflozin (7%). EuDKA was most commonly found in patients using canagliflozin (100%, n = 3), followed by empagliflozin (77.7%) and Dapagliflozin (48.3%). However, no statistically significant difference was found in the development of EuDKA with any particular type of SGLT2i after multiple linear regression. MICU admission rate was 66.6% in patients on canagliflozin, 45.1% in Dapagliflozin, and 33.3% in the empagliflozin group. In-hospital mortality was only noted in the dapagliflozin group (3.2% OR 0.293 CI 0.01–7.7). The median length of stay in canagliflozin, Dapagliflozin, and empagliflozin was 4 (IQR 3–4.5), 5 (3–9), and 9 (IQR 2–16) days, respectively. Median DKA duration was 2 (IQR 2–2.5) in canagliflozin, 3 (2–4) in Dapagliflozin, and 2 (2–3) days in the empagliflozin group. Median pH was 7.17 (IQR 7.17–7.22) in canagliflozin, 7.21 (IQR 7.21–7.31) in dapagliflozin, and 7.29 (IQR 7.29–7.34) in the empagliflozin group [Table 2].

**Table 2:** Demographics based on SGLT2i class.

Characteristics	Units	Canagliflozin	Dapagliflozin	Empagliflozin
Total T2D patients on SGLT2i	N, %	14	7280	2646
Prevalence of total DKA	N, %	3/14 (0.21%)	31/7280 (0.43%)	9/2646 (0.34%)
Prevalence of EuDKA	N, %	3/14 (0.21%)	15/7280 (0.21%)	7/2646 (0.26%)
Prevalence of hDKA	N, %	0	16/7280 (0.23%)	2/2646 (0.08%)
Males	N	1	15	3
Females	N	2	16	6
**Ethnicities**	N			
MENA		2	23	7
Southeast Asia		1	4	1
Others		0	4	1
**Triggers:**	N			
Gastroenteritis		1	8	2
Psychiatric disorder		0	2	0
Pancreatitis		0	2	0
Surgery		0	0	1
Insulin non-compliance		0	3	1
Infections		2	10	2
**Diabetes complications**	N			
Retinopathy		0	4	2
Nephropathy		1	4	2
Diabetic foot		0	0	2
Amputation		0	0	1
Peripheral arterial disease		0	0	2
**Co-morbidities**	N			
Coronary artery disease		1	5	2
Heart failure		2	1	1
Asthma		0	8	3
Chronic liver disease		0	1	0
Hypertension		1	19	3
Malignancy		0	1	1
Insulin use	N	3	20	6
Sulfonylurea	N	2	10	3
Metformin	N	1	21	6
Thiazolidinediones	N	0	10	5
Meglitinides	N	0	1	1
Alpha-glucosidase inhibitor	N	0	1	0
GLP-1 agonist	N	1	3	1
DPP-4 inhibitors	N	0	12	5
Inhaled steroids	N	0	8	3
In-hospital Mortality	N	0	1	0
MICU admission	N	2	14	3
HbA1c	mmol/mol	96.7	79.2	72.7
HbA1c, Median (IQR)	Percentage	11 (11–11.6)	9.4 (7.95–11.5)	8.8 (8.7–10.6)
Admission Glucose, Median (IQR)	mmol/L	9.6 (8.25–9.6)	14.4 (10.3–22.4)	12.2 (10.8–13.7)
pH, Median (IQR)	NA	7.17 (7.17- 7.22)	7.21 (7.21 -7.31)	7.29 (7.29—7.34)
Anion gap, Median (IQR)	mEq/L	25.5 (21.8–26.8)	19 (17.5–21.3)	20 (17–23)
Length of stay, Median (IQR)	Days	4 (3–4.5)	5 (3–9)	9 (2–16)
DKA duration, Median (IQR)	Days	2 (2–2.5)	3 (2–4)	2 (2–3)

## Discussion

This study represents the first comprehensive evaluation of the prevalence and determinants of EuDKA in a cohort of T2D patients exposed to SGLT2i. In a population of 9940 T2D patients taking SGLT2i during five years (2015–2020), 43 developed DKA. In this cohort, we found the point prevalence of EuDKA at 58.1% amongst T2D patients within our cohort. Previously a point prevalence of 69.8% for EuDKA in T2D and T1D combined is reported by Clark et al.^13^. Our cohort excluded T1D patients because the FDA does not recommend SGLT2i use in this population^16^. Additionally, we found infections and insulin non-adherence as the key drivers to development and presentation with EuDKA.

In our study, precipitators of EuDKA were similar to previous studies, with a notable exception of fasting, which was not found in any of our patients who developed EuDKA [Table 1]. We did not find any previously known precipitator in 22 patients. As discussed by Clark et al., we also think that SGLT2i use could have led to DKA in these patients in the absence of additional triggers^13^. However, there is a possibility of other precipitating factors that could not be assessed in our study. The median length of stay (LOS) in our study varied from 4 to 9 days, depending upon the type of SGLT2i. Variable LOS is reported before, ranging from 3.53 to 6 days^13,17^. The median time to close the anion gap was 2–3 days, based on the type of SGLT2i used, which is almost double compared to the previous^13,17^.

In a review of 101 case reports, the highest number (63 cases) of DKA was seen in patients taking canagliflozin, reflected in our study. Hence, Canagliflozin is probably most strongly associated with the development of EuDKA^18^. We also reviewed and tabulated data from the latest 30 case reports describing EuDKA secondary to SGLT2i in T2D patients^19–48^ [Table 3]. Our study showed a slightly higher prevalence of EuDKA in females, which was not statistically significant [Table 1]. Previous retrospective study and our review of the last 30 case reports showed a slight male predominance^13^ [Table 3]. Reviews that have been solely on case reports have shown a considerable difference with 75% prevalence in females^49^. Fasting has been reported as the most common precipitator in the case reports (47.5%), whereas it was 14% in the previous descriptive study on 43 patients (including 25 with T2D) and 0% in our study. Analysis of case reports is relevant in rare diseases in the absence of more extensive studies. However, any conclusions drawn from case reports must be reconsidered when data from more extensive studies with a more representative cohort becomes available.

**Table 3:** Literature review of last 30 reported cases of EuDKA in type 2 diabetes mellitus patients on SGLT2i.

Clinical characteristics	Units	*N* = 40
Age (Mean ± SD)	Years	61.3 (± 12.4)
**Gender**	N (%)	
Females		18 (45%)
Males		22 (55%)
BMI (Mean ± SD)	Kg/m^2^	27.9 (± 3.83)
Glucose upon admission (Mean ± SD)	mmol/L	9.33 (± 2.98)
PH (Mean ± SD)	NA	7.22 (± 0.15)
Anion Gap (Mean ± SD)	mEq/L	22.6 (± 5.41)
Lactate (Mean ± SD)	mmol/L	1.4 (1.2–2.1)
Creatinine (Mean ± SD)	umol/L	95.4 (88.4–118)
WBC (Mean ± SD)	10^3^/uL	12.9 (± 4.09)
HbA1c	mmol/mol	66.1
HbA1c, Median (IQR)	Percentage	8.2 (7.07–9.55)
**Triggering factors**	N (%)	
Fasting		19 (47.5%)
Infection		5 (12.5%)
Pancreatitis		2 (5%)
Surgery		1 (2.5%)
Ketogenic Diet		1 (2.5%)
Myocardial Infarction		1 (2.5%)
Drugs (NSAIDs)		1 (2.5%)
Malignancy		2 (5%)
Insulin non-compliance		0
Unknown triggers		8 (20%)
**SGLT 2 inhibitors type**	N (%)	
Empagliflozin		25 (62.5%)
Dapagliflozin		11 (27.5%)
Canagliflozin		1 (2.5%)
Tofogliflozin		2 (5%)
Unknown		1 (2.5%)
Current Insulin use	N (%)	12 (30.8%)
Sulfonylurea	N (%)	6 (15.4%)
Metformin	N (%)	32 (82.1%)
Thiazolidinediones	N (%)	5 (12.8%)
Meglitinides	N (%)	3 (7.7%)
Alpha-glucosidase inhibitors	N (%)	0
GLP-1 agonist	N (%)	0
DDP-4 inhibitors	N (%)	7 (17.9%)
Antipsychotics	N (%)	2 (5.1%)
Steroids	N (%)	0
EuDKA duration, Median (IQR)	Days	2 (1–2)
Hospital days, Median (IQR)	Days	14 (6–25)
Need for admission to ICU	N (%)	12 (32.4%)
In-hospital mortality	N (%)	0

EuDKA and hDKA have different pathophysiologies. In EuDKA, there is a relatively milder intensity of insulin deficiency and resistance. Moreover, urinary glucose clearance in EuDKA is double compared to hDKA (estimated around 0.3 ml/min/kg in hDKA and 0.6 ml/min/kg in EuDKA)^50^. Considering distinct pathophysiologies, differences in precipitators, presentations, clinical course, and outcomes can be expected. To evaluate this, we compared patient demographics, presentation, clinical course, and outcomes in patients with EuDKA and hDKA. To the best of our knowledge, there are no prior studies comparing EuDKA and hDKA in the T2D population taking SGLT2i. Our study found some key clinical differences. Firstly, EuDKA was probably more prevalent in females (60%), whereas hDKA did not seem to have a gender preference. Secondly, the median RBS in EuDKA and hDKA differed as expected (10.3 vs. 21.8 mmol/l). Among the precipitators, surgery seemed to be more associated with the development of EuDKA as compared to hDKA, and insulin non-compliance was probably more common in hDKA compared to EuDKA. Among the SGLT2i types, Dapagliflozin was probably the most commonly associated with both types of DKA, followed by Empagliflozin and Canagliflozin (which seemed to be mainly associated with EuDKA as none of our T2D patients on Canagliflozin developed hDKA). ICU admission rates were comparable in both groups. However, mortality seemed to be more associated with EuDKA, although our sample size was not powered to analyze mortality. Although many clinical differences among the two groups exist, we only found statistically significant differences in rates of infections, diabetic retinopathy, fasting, and random blood glucose levels. A more extensive comparison between EuDKA and hDKA in T2D patients taking SGLT2i with larger sample sizes can validate our results.

The phenomenon of EuDKA has been reported in the literature as early as 1989^51^. However, with the advent and success of SGLT2i in T2D management, it has become a focus of discussion in diabetology. Although it is a rare phenomenon, the scale of use of SGLT2i has led to an increasing number of cases of EuDKA. Throughout the years, its definition has evolved. The current definition of EuDKA is the presence of ketoacidosis with a pH ≤ 7.3, bicarbonate ≤ 15 mmol/l, anion gap > 12 mmol/l in the setting of lower-than-expected glucose level (RBS < 14 mmol/l)^13^. SGLT2i use is increasing due to its positive effects on glycaemic control, body weight, heart failure exacerbations, and cardiovascular deaths^52,53^.

The mechanism of EuDKA secondary to SGLT2i is not yet fully understood. Perry RJ et al. conducted a study on healthy and T2D rats given SGLT2i. They concluded that low insulin levels and dehydration promote EuDKA by raising serum catecholamine and corticosterone levels, leading to increased white adipose tissue lipolysis^54^. A similar mechanism has been proposed in humans with propositions that euglycemia occurs in the setting of SGLT2i associated DKA when the renal glucose clearance exceeds endogenous glucose production^55,56^.

Our study’s principal strength lies in its novelty in carrying out the first comprehensive exploration of the distribution, determinants, and other factors impacting the outcomes of T2D patients with EuDKA.

Our study has some limitations inherent when reviewing such data schemes. Firstly, its retrospective design meant difficulties were encountered with missing values and case adjudication, especially those of precipitating factors. Secondly, owing to its low incidence, our sample size was small; a bigger sample size could improve our results’ validity and generalizability. A more extensive prospective study on T2D patients on SGLT2i may yield more reliable results regarding risk factors and pathophysiology of EuDKA in this population.

## Conclusion

In our cohort of T2D patients taking SGLT2i, the prevalence of DKA is around 0.43%, with a prevalence of EuDKA and hDKA 0.25% and 0.18%, respectively. Overall, DKA (including EuDKA) was primarily triggered by infection. Additionally, patients with more complicated diabetes are probably prone to develop EuDKA compared to hDKA. The differential contribution of specific SGLT2i analogs remains uncertain and will need examination by larger sample-sized prospective studies.

## Supplementary Information

Supplementary Information 1.

Supplementary Information 2.

Supplementary Information 3.

Supplementary Information 4.

Supplementary Information 5.

## Data Availability

Available upon request.

## Abbreviations

EuDKA: Euglycemic diabetic ketoacidosis
hDKA: Hyperglycaemic DKA
T2D: Type 2 diabetes mellitus
T1D: Type 1 diabetes mellitus
SGLT2i: Sodium-glucose co-transporter-2 inhibitors
MICU: Medical intensive care unit
GLP-1: Glucagon-like peptide 1 receptor
DPP-4: Dipeptidyl peptidase-4
RBS: Random blood sugar
BMI: Body mass index
POC: Point of care
LOS: Length of stay

## Code availability

Available upon request.
