# Supplementary material for: SGLT-2 inhibitors associated euglycemic and hyperglycemic DKA in a multicentric cohort
Source: Sci Rep. 2021 May 13;11:10293. doi: 10.1038/s41598-021-89752-w (PMC8119406; doi:10.1038/s41598-021-89752-w)
Supplement: Supplementary file 1 — Supplementary Information 1. [file 41598_2021_89752_MOESM1_ESM.pdf]

One-Way ANOVA (Welch's)

	F	df1	df2	p
Age	2.72002	1	34.0	0.108
Height	3.22966	1	24.3	0.085
Weight	0.53995	1	41.0	0.467
BMI	0.15719	1	39.4	0.694
Glucose upon admission	50.45048	1	19.9	< .001
Temperature at admission	1.45263	1	24.1	0.240
HBA1C	0.94230	1	33.2	0.339
WBC (admission)	0.52185	1	25.8	0.477
HGB (admission)	0.75092	1	40.9	0.391
PLT (at admission)	3.51156	1	32.1	0.070
Fasting glucose	6.16518	1	18.9	0.023
Creatinine (admission)	0.28920	1	28.9	0.595
Albumin	0.89178	1	33.0	0.352
Lactate	0.00513	1	29.1	0.943
PH	0.33947	1	27.0	0.565
Anion gap	0.08533	1	40.3	0.772
DKA duration (Days)	0.03343	1	38.6	0.856
Q sofa score if MICU admission	0.13303	1	27.6	0.718
