# Supplementary material for: SGLT-2 inhibitors associated euglycemic and hyperglycemic DKA in a multicentric cohort
Source: Sci Rep. 2021 May 13;11:10293. doi: 10.1038/s41598-021-89752-w (PMC8119406; doi:10.1038/s41598-021-89752-w)
Supplement: Supplementary file 2 — Supplementary Information 2. [file 41598_2021_89752_MOESM2_ESM.pdf]

Kruskal-Wallis

	χ^2	df	p
Gender	0.54556	1	0.460
Ethnicity	0.80503	1	0.370
BP at admission	0.00546	1	0.941
Infections	7.28368	1	0.007
Psychiatric disorder	2.84553	1	0.092
DM retinopathy	0.20348	1	0.652
DM nephropathy	2.55148	1	0.110
Diabetic foot	0.05577	1	0.813
Amputation	1.38889	1	0.239
CAD	0.13228	1	0.716
PAD	1.47512	1	0.225
COPD	NaN	1	NaN
Heart failure	0.50316	1	0.478
Liver disease	1.38889	1	0.239
HTN	0.18566	1	0.667
Active cancer	0.72000	1	0.396
Hematologic cancer	0.72000	1	0.396
Albuminuria	1.76174	1	0.184
SGLT-2 Inhibitor	0.19280	1	0.661
Current insulin use	0.31471	1	0.575
Current insulin type	1.00526	1	0.316
Current insulin dose	2.58547	1	0.108
Complaint to insulin	1.59781	1	0.206
Sulfonylurea	0.67222	1	0.412
Metformin	1.03200	1	0.310
Thiazolidinediones	0.21356	1	0.644
Meglitinides	2.84553	1	0.092
a glucosidase inhibitors	1.38889	1	0.239
GLP-1 agonist	0.00786	1	0.929
DPP IV inhibitors	0.30492	1	0.581
Corticosteroids	0.07663	1	0.782
Antipsychotic	NaN	1	NaN
Hospital days	0.86243	1	0.353
In-hospital mortality	0.72000	1	0.396
Out of hospital mortality	0.72000	1	0.396
Need for MICU admission	8.19e-4	1	0.977
