# Supplementary material for: SGLT-2 inhibitors associated euglycemic and hyperglycemic DKA in a multicentric cohort
Source: Sci Rep. 2021 May 13;11:10293. doi: 10.1038/s41598-021-89752-w (PMC8119406; doi:10.1038/s41598-021-89752-w)
Supplement: Supplementary file 3 — Supplementary Information 3. [file 41598_2021_89752_MOESM3_ESM.pdf]

Linear Regression

Model Fit Measures

Model	R	R^2	Adjusted R^2
1	0.809	0.655	0.495

Omnibus ANOVA Test

	Sum of Squares	df	Mean Square	F	p
Fasting glucose	391.99	1	391.99	8.0446	0.014
DM retinopathy	343.95	1	343.95	7.0587	0.020
SGLT-2 Inhibitor	2.88	2	1.44	0.0296	0.971
Compliance to insulin	24.61	1	24.61	0.5051	0.490
Infections	257.24	1	257.24	5.2792	0.039
Residuals	633.45	13	48.73		

Note. Type 3 sum of squares

Model Coefficients - Glucose upon admission

Predictor	Estimate	SE	t	p
Intercept [a]	4.50	11.188	0.402	0.694
Fasting glucose	1.52	0.535	2.836	0.014
DM retinopathy:				
Yes – No	19.89	7.485	2.657	0.020
SGLT-2 Inhibitor:				
Dapagliflozin – Canagliflozin	-1.69	7.637	-0.221	0.829
Empagliflozin – Canagliflozin	-1.09	8.000	-0.136	0.894
Compliance to insulin:				
Yes – No	-5.68	7.990	-0.711	0.490
Infections:				
Yes – No	7.87	3.427	2.298	0.039

[a] Represents reference level

Assumption Checks

Collinearity Statistics

	VIF	Tolerance
Fasting glucose	1.13	0.887
DM retinopathy	1.05	0.957
SGLT-2 Inhibitor	1.10	0.906
Compliance to insulin	1.12	0.896
Infections	1.08	0.930

Q-Q Plot

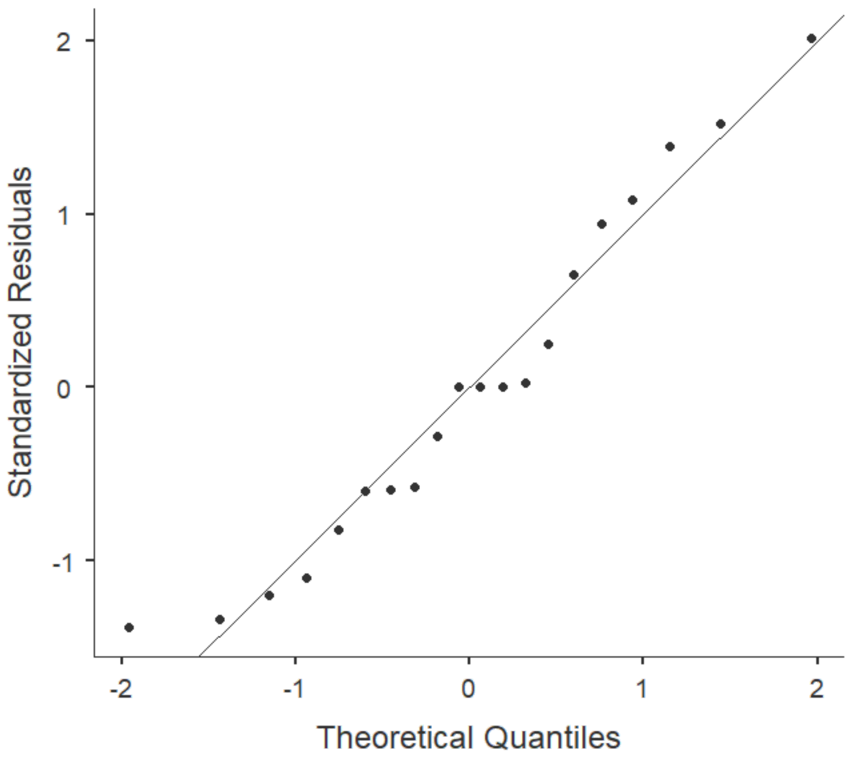
2
1
0
-1
Standardized Residuals
-2
-1
0
1
2
Theoretical Quantiles
