# Supplementary material for: SGLT-2 inhibitors associated euglycemic and hyperglycemic DKA in a multicentric cohort
Source: Sci Rep. 2021 May 13;11:10293. doi: 10.1038/s41598-021-89752-w (PMC8119406; doi:10.1038/s41598-021-89752-w)
Supplement: Supplementary file 5 — Supplementary Information 5. [file 41598_2021_89752_MOESM5_ESM.docx]

**Table 4: Bivariate pairwise comparison between EuDKA and hDKA patients**

| **Variable** | **Statistic** | **p** | **Mean** | **SE** | **95% CI** | |
| --- | --- | --- | --- | --- | --- | --- |
|  |  |  |  |  | **Lower** | **Upper** |
| Q sofa score if MICU admission | -0.389 | 0.699 | -0.0844 | 0.217 | -0.523 | 0.354 |
| Albumin | 0.835 | 0.409 | 4.1176 | 4.933 | -5.846 | 14.081 |
| Fasting glucose | -2.639 | 0.013 | -3.2444 | 1.229 | -5.763 | -0.726 |
| Age | -1.645 | 0.108 | -6.4889 | 3.944 | -14.454 | 1.476 |
| BMI | 0.389 | 0.699 | 0.8167 | 2.098 | -3.421 | 5.054 |
| Temperature at admission | -1.322 | 0.194 | -0.2842 | 0.215 | -0.719 | 0.150 |
| HBA1C | -0.996 | 0.325 | -0.6004 | 0.603 | -1.818 | 0.617 |
| Glucose upon admission | -8.111 ᵃ | < .001 | -12.9913 | 1.602 | -16.226 | -9.757 |
| WBC (admission) | -0.781 ᵃ | 0.439 | -1.5904 | 2.037 | -5.703 | 2.522 |
| HGB (admission) | 0.815 | 0.420 | 0.6062 | 0.744 | -0.896 | 2.109 |
| PLT (at admission) | -1.937 | 0.060 | -57.2200 | 29.547 | -116.892 | 2.452 |
| Creatinine (admission) | 0.466 | 0.644 | 32.8193 | 70.441 | -109.439 | 175.077 |
| Lactate | 0.623 | 0.537 | 0.6350 | 1.019 | -1.429 | 2.699 |
| PH | -0.500 | 0.620 | -0.1017 | 0.203 | -0.512 | 0.309 |
| Anion gap | 0.282 | 0.779 | 0.5889 | 2.085 | -3.621 | 4.799 |
| Current insulin dose | -0.505 | 0.616 | -2.8235 | 5.591 | -14.122 | 8.475 |
| Hospital days | 1.405ᵃ | 0.168 | 6.3289 | 4.504 | -2.768 | 15.426 |
| Height | -1.566 ᵃ | 0.125 | -20.1765 | 12.886 | -46.200 | 5.846 |
| Weight | 0.695 | 0.491 | 4.1222 | 5.930 | -7.854 | 16.098 |
| DKA duration | -0.180 | 0.858 | -0.0733 | 0.407 | -0.895 | 0.749 |
|  |  |  |  |  |  |  |

ᵃLevene’s test is significant (p < .05), suggesting a violation of the assumption of equal variances.

ᵇ One or both groups do not contain enough observations.

**Table 5: Bivariate pairwise comparison between EuDKA and hDKA patients**

| **Variables** | **Value** | **df** | **p-value** |
| --- | --- | --- | --- |
| Gender | 0.424 | 1 | 0.515 |
| Ethnicity | 9.1 | 10 | 0.522 |
| Psychiatric disorder | 2.91 | 1 | 0.088 |
| **DM complications**  Diabetic retinopathy  Diabetic foot  Amputation  CAD  PAD  Microalbuminuria | 0.208  0.057  1.42  3.01  1.51  1.80 | 1  1  1  2  1  1 | 0.648  0.811  0.233  0.222  0.219  0.179 |
| **Co-morbidities**  HF  Liver disease  HTN  Active cancer | 0.515  1.42  0.190  0.737 | 1  1  1  1 | 0.471  0.233  0.663  0.391 |
| Type of SGLT2i | 4.80 | 2 | 0.091 |
| Current insulin use | 0.322 | 1 | 0.570 |
| **Medications**  Sulfonylureas  Metformin  Thiazolidinediones  Meglitinides  Alpha-glucosidase inhibitors  GLP-1 agonists  DPP-4 inhibitors  Corticosteroids | 0.688  1.25  0.219  2.91  1.42  0.08  0.312  0.078 | 1  2  1  1  1  1  1  1 | 0.407  0.534  0.640  0.088  0.233  0.920  0.576  0.779 |
| **Precipitating factors**  Infections  Insulin non-compliance  Pancreatitis  Surgery | 7.46  1.88  0.057  0.737 | 1  1  1  1 | 0.006  0.170  0.811  0.391 |
| **Outcomes**  In-hospital mortality  Need for MICU admission | 0.737  8.38 * 10^-4^ | 1  1 | 0.391  0.977 |
